# Higher senescence associated secretory phenotype and lower defense mediator in urinary extracellular vesicles of elders with and without Parkinson disease

**DOI:** 10.1038/s41598-021-95062-y

**Published:** 2021-08-04

**Authors:** Shu-hui Yeh, Chia-Hsueh Lin, Yun-Jung Yang, Li-Wei Lin, Chih-Wen Tseng, Kuender D. Yang

**Affiliations:** 1 Institute of Long Term Care, Mackay Medical College, New Taipei City, 252 Taiwan; 2 Department of Medical Research, Mackay Memorial Hospital, Taipei, 104 Taiwan; 3 Institute of Clinical Sciences, National Yang Ming University, Taipei, 112 Taiwan; 4 Institute of Clinical Sciences, National Yang-Ming Chiao-Tung University, Taipei, 112 Taiwan; 5 Department of Nursing, Huang Kuang University, Taichuang, 433 Taiwan; 6 Department of Microbiology and Immunology, National Defense Medical Center, Taipei, 114 Taiwan

**Keywords:** Biological techniques, Biomarkers, Health care

## Abstract

Youth fountain and aging culprits are usually sought and identified in blood but not urine. Extracellular vesicles (EVs) possess parental cell properties, circulate in blood, CSF and urine, and provide paracrine and remote cell–cell communication messengers. This study investigated whether senescence‐associated secretory phenotype (SASP) and immune defense factors in EVs of urine could serve as biomarkers in elderly individuals with and without a comorbidity. Urine samples from young adults and elderly individuals with and without Parkinson disease (PD) were collected and stored at − 80 °C until studies. Urine EVs were separated from a drop-through solution and confirmed by verifying CD9, CD63, CD81 and syntenin expression. The EVs and drop-through solution were subjected to measurement of SASP cytokines and defense factors by Milliplex array assays. Many SASP cytokines and defense factors could be detected in urinary EVs but not urinary solutions. Elderly individuals (age > 60) had significantly higher levels of the SASP-associated factors IL-8, IP-10, GRO, and MCP-1 in EVs (*p* < 0.05). In contrast, some defense factors, IL-4, MDC and IFNα2 in EVs had significantly lower levels in elderly adults than in young adults (age < 30). Patients with and without PD exhibited a similar SASP profile in EVs but significantly lower levels of IL-10 in the EVs from patients with PD. This study used a simple device to separate urinary EVs from solution for comparisons of SASP and defense mediators between young adults and elders with and without PD. Results from this study indicate that aging signature is present in EVs circulating to urine and the signatures include higher inflammatory mediators and lower defense factors in urinary EVs but not solutions, suggesting a simple method to separate urinary EVs from solutions for searching aging mechanistic biomarkers may make prediction of aging and monitoring of anti-senolytic interventions possible.

## Background

Aging has been referred to as the accumulation of senescent cells, resulting in a proinflammatory pattern called the senescence‐associated secretory phenotype (SASP) occurring in circulation and in tissues^1^. Aging also involves immunosuppression and is associated with a higher susceptibility and fatality to emerging infections such as influenza^2^ and the SARS-CoV-2 pandemic^3^. There are many trigger factors leading to cellular senescence, such as oxidative stress, telomere shortening and/or DNA damage, that induces the SASP of the aging process. Oxidative stress and/or DNA damage activates nuclear factor kappa B (NFκB) and p38 kinase phosphorylation for the induction of SASP-associated factors, including IL-1β, IL-6, CXCL8 (IL-8), MCP-1, IP-10, CCL-2, VEGF, and/or CXCR2, resulting in senescence in different types of cells. SASP-associated factors have been shown to cause degenerative diseases in elderly individuals^4^, and age-related poor defense mediators, such as lower concentrations of interferons, contribute to the susceptibility of infections^5^. Many senescent biomarkers have been identified in tissues and blood but not in urine^1,4,6^. Traditionally, we performed urine analysis by detecting cells (5–15 µm) and molecules such as protein, sugar and nitrite (< 30 nm) to assess physiological and pathological situations^7^. Urinary EVs possessing parental cell properties, providing paracrine and remote cell–cell communication messengers, and circulating in blood, CSF and urine may contain biomarkers of young and/or old imprints. This study postulated that age-related SASP factors and immune defense factor(s) are present in urinary EVs but not urinary solutions presenting unique biomarkers for the prediction of elderly patients with and without comorbidity.

Extracellular vesicles including exosomes which possess lipid bilayers with size between 50 and 150 nm provide cell–cell communication messengers of protein, RNA and DNA. EVs can be released into body fluids by most cell types, including stem cells, cancer cells or cells under stress^8–10^. Characteristically, EVs share similar cell surface components, such as expression of tetraspanins (CD9, CD63, and/or CD81), and different intravesicular components of immune mediators, as well as miRNA and proteins derived from different origins depending on cell type. For instance, circulating EVs with high miRNA21 levels have been implicated in colon, pancreas or breast cancer progression^11^, and maternal circulating EVs with abnormal protein or miRNA profiles have been correlated with preeclampsia^12^. Abnormal surface glycosphingolipid expression of neuron EVs has also been implicated in the accumulation of beta-amyloid deposition associated with Alzheimer’s disease^13^. These biomarkers are usually detected in blood or CSF but not urine.

Extracellular vesicles are heterogenous vesicles in size and contents so that it is a challenge to isolate the representative urinary EVs for studies of biomarkers. Classically, EVs are isolated by combined separation techniques depending on individual situations (e.g. large amount, small amount, complexity or simplicity of tissue fluids) and advantageous skills or devices for rapid test or precision test^14,15^. Recently, efficient ultrafiltration-based protocols to isolate or deplete EVs have been made possible^16,17^, including isolation of urinary EVs^18^. We have recently revealed that EVs with sizes between 30 and 200 nm isolated by a series of filtrations carry beneficial signals for neurotrophic and/or anti-inflammatory signals in a neuropathic pain model^19,20^. Given the less complexity and large amount of urine, we designed a series of filtrations from depleting cell debris above 1 µm, cutting off apoptotic vesicles above 0.22 µm to isolating EVs between 0.22 and 0.03 µm. The urinary EVs isolated were washed 3 times and subjected to comparisons of SASP levels among young adults and old adults without and with PD after confirming the size, particles and exosomal markers. We postulated that the novel method to separate urinary EVs from urinary solutions will identify the SASPs in urinary EVs as aging biomarker. This study investigated whether different SASPs and defense factors are present in urinary EVs, but not urinary solutions, among young and elderly individuals with and without PD.

## Methods

### Study design and subjects

This study was designed to recruit normal elderly individuals over 60 years of age participating in the geriatric day care center and adult volunteers about 30 years of age. The elderly [n = 10, age 69 ± 6.0 (mean ± SD)], young volunteers [n = 10, age 26 ± 8.0 (mean ± SD)] and patients with PD [n = 24, age 67 ± 7 (mean ± SD)] from a sick friend association of PD were enrolled after informed consent was obtained. The 24 KD patients recruited from two sick friend associations are not homogenous in the severity or stages but definite diagnosis. The demographic data are summarized in Table 1. All experiments were performed in accordance with relevant guidelines/regulations of the institution review board at Mackay Memorial Hospital.

**Table 1:** Demographic data of the subjects studied.

Young adults	Conditions	Age	Sex
**(a) Demographic data of young adults**			
Y1	Normal	19	F
Y2	Normal	19	M
Y3	Normal	20	M
Y4	Normal	20	F
Y5	Normal	21	F
Y6	Normal	21	M
Y7	Normal	24	F
Y8	Normal	37	F
Y9	Normal	38	F
Y10	Normal	38	F
Mean (SD)		26 (8)	

Old adults	Comorbidities	Age	Sex
**(b) Demographic data of old adults**			
O1	Hypertension	61	M
O2	Normal	62	F
O3	Type 2 diabetes	63	M
O4	Normal	65	F
O5	Normal	68	F
O6	Normal	68	M
O7	Normal	71	F
O8	Normal	74	M
O9	Normal	76	M
O10	Normal	78	F
Mean (SD)		69 (6)	

PD patients	Co-morbidities	Age	Sex
**(c) Demographic data of PD patients**			
PD1	PD	52	M
PD2	PD, Depression	57	F
PD3	PD, Depression	58	M
PD4	PD, Urinary symptom	60	M
PD5	PD	60	M
PD6	PD	61	F
PD7	PD	62	M
PD8	PD, Type 2 diabetes	63	F
PD9	PD	63	M
PD10	PD, Hypertension	64	M
PD11	PD, Hearing loss	65	F
PD12	PD	65	F
PD13	PD, Hypertension	67	F
PD14	PD	68	F
PD15	PD, Type 2 diabetes	68	M
PD16	PD, Urinary symptom	70	M
PD17	PD, Hypertension	70	M
PD18	PD	70	M
PD19	PD, Type 2 diabetes	71	F
PD20	PD	72	M
PD21	PD, Osteoarthritis	75	F
PD22	PD	76	F
PD23	PD	77	M
PD24	PD	82	F
Mean (SD)		67 (7)	

*PD* Parkinson disease, *M* male, *F* female, *SD* standard deviation.

### Sample collection

Urine samples (40 ml) were collected in the morning while young volunteers and elderly individuals were participating in the day care center activities. For collecting samples from patients with PD, urine (40 ml) was collected in the morning before they began participating in the day care center activities. These urine samples were collected into a conical tube and stored at − 80 °C within 2 h until studies were performed.

### Separation of urinary EVs and urinary solutions

**Figure 1:**
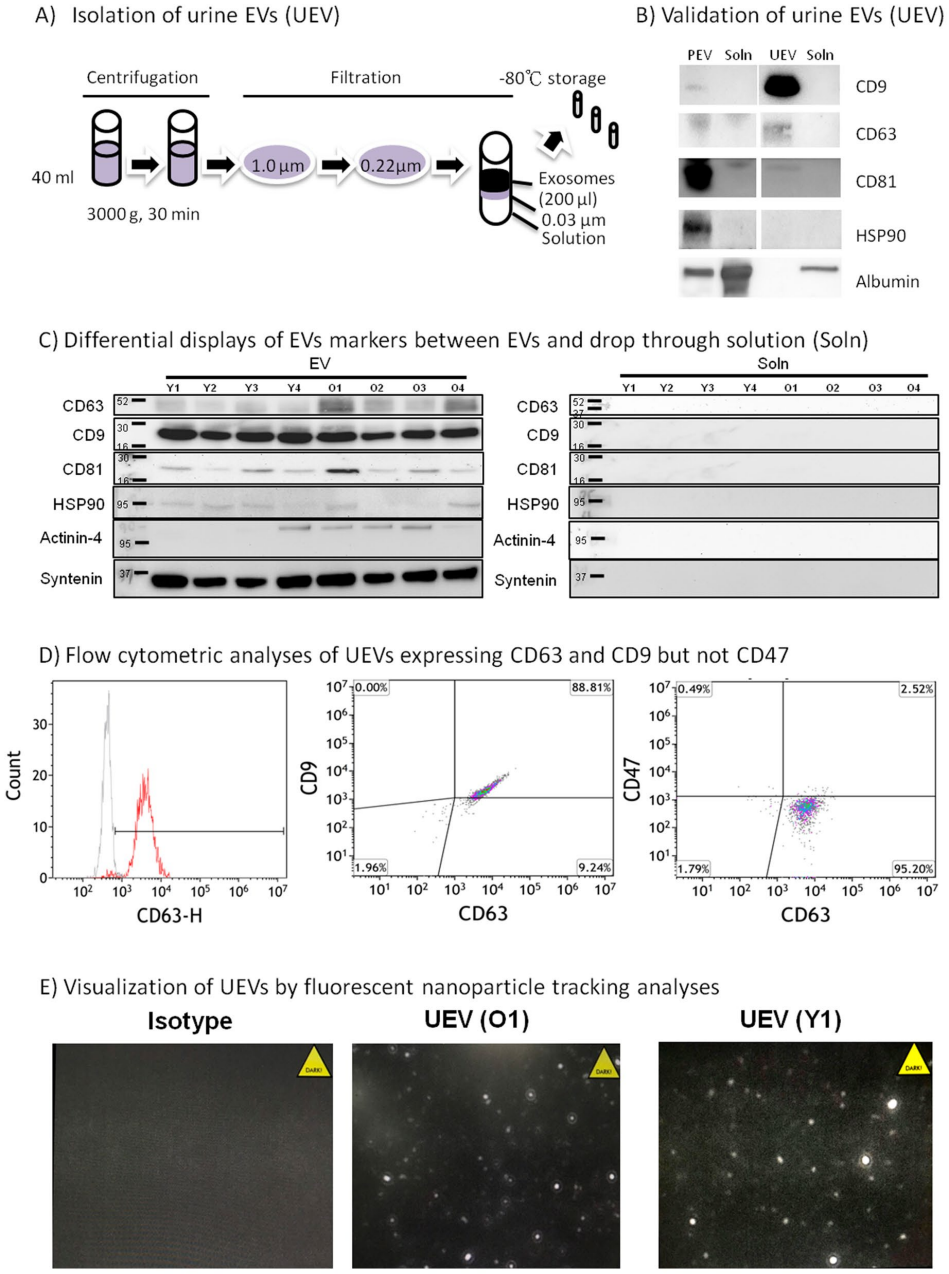
Isolation and validation of urine EVs. Urine (40 ml) was centrifuged to deplete cell debris, followed by depletion of apoptotic bodies by 1.0 µm and 0.22 µm filters, and finally to isolate EVs from solution by another 0.03 µm filter (A). Urine EVs (UEV) showed a prominent CD9 expression in contrast to plasma EVs (PEV) expressing a higher CD81, and no exosomal markers (CD9, CD63, CD81 or HSP90) was detected in the drop through solution (Soln) in a normal adult. The PEV isolated from the young adult (Y1) by ExoQuick contained some albumin contamination, but UEV, isolated from the young adult (Y1) by a series of membrane filtrations as shown in (**A**), contained neglectable albumin contamination (**B**). Further studies showed that similar levels of CD9/CD81/HSP90/syntenin expression were found in the EVs from young adults (n = 4; Y1, Y2, Y3 and Y4) and the EVs from old adults (n = 4; O1, O2, O3 and O4), but some extent of higher CD63/actinin-4 expression in the EVs from the elderly (**C**). The blots presented here are derived from the images of original western blots which are shown in Supplementary Fig. 1B and C. The flow cytometric analyses of CD63, CD9 and CD47 expression using anti-CD63-beads enriched UEVs showed that CD9 co-expressed with CD63, but not CD47, on UEVs in three replicable experiments (**D**). We also performed fluorescent nanoparticle tracking analyses (NTA), gating the size between 30 and 300 nm, to visualize the UEVs derived from old (O1) and young (Y1) adults in three replicable experiments (**E**).

In the studies, the urine samples (40 ml) were rapidly thawed in a 37 °C water bath, which was followed by centrifugation at 4 °C at 3000 g for 30 min, and passing through a 1.0 µm filter, made in polyethersulfone (PES) membrane, to remove cell debris. The samples were then passed through a 0.22 µm filter, made in PES membrane, to separate apoptotic bodies from EVs and urine solutions, and finally, the EV and solution fractions were separated by the other 0.03 µm filter, made in regenerated cellulose (RC) membrane (Fig. 1A). The EVs retained on the filter were washed using PBS for 3 times, and the final concentration was adjusted to 200-fold (40 ml input of urine and 200 µl output of EVs). The output 200 µl EVs were separated into 10 µl (10 vials) and 25 µl (4 vials) and stored at − 70 °C until studies. The EV samples were subjected to membrane solubilization by treatment with radioimmunoprecipitation assay (RIPA) buffer at a 1:4 proportion before measurement of total protein by bicinchoninic acid (BCA) assay (Thermo Fisher Sci., USA). The protein in the drop-through solution was also measured by the BCA kit. In addition, we isolated EVs from plasma (PEV) by ExoQuick (System Biosciences Inc., Palo Alto) as manufactural recommendation for the comparison of EV surface characters between PEV and urine EVs (UEV). In brief, the plasma 500 µl was incubated with 126 µl reagent without agitation for 30 min at 4 °C. After incubation, the reaction tube was centrifuged at 1500×*g* for 30 min, and the precipitated EVs in pellet was resuspended in 100 µl using sterile PBS, and stored in aliquots at − 80 °C until studies. The reason we used commercial ExoQuick kit to isolate PEV is because the high complexity and low volume of plasma is not suitable for the isolation by a series of filtrations. In contrast, urine samples are less complexity and large volume suitable for the isolation by a series of filtrations and washes. The PEV from a young adult who donated the first urine sample was isolated by ExoQuick and used as a comparison control of the UEV from the same donor isolated by a series of filtrations for the confirmation of EVs characters using Western blots, NTA and flow cytometry described below.

### Number and size of urine EVs by a nanoparticle tracking analyzer (NTA)

A nanoparticle tracking analyzer (NTA) has been previously used to analyze EVs of urine derived from mice^21^. We used NanoSight LM10 (Marvern Panalytical, UK) to measure size, number and fluorescent contour of EVs because the NanoSight NTA could precisely estimate the size of EVs better than other devices^22^. For measurement, concentrated urine EVs were diluted 1:1000 and placed on ice before being applied to the sample chamber of the NTA. We focused on analyses of vesicles size between 30 and 300 nm. The NTA measurement for each sample was collected in triplicate. Data regarding the vesicle size vs. concentration curve in the 30–300 nm range were acquired in all the samples studied, and the number of EVs in each sample is used for normalization of the protein and mRNA of the SASP in EVs for comparisons between young and old adults. To visualize the UEVs, we also performed fluorescent NTA analyses, which is known to better delineate the contour of EVs by gating the size between 30 and 300 nm^23^, by using the antibody directed against CD63 labeled with AF488.

### Determination of differential markers between urine EVs and fractions

The same amounts (20 µg) of protein derived from young volunteers and elderly individuals were subjected to protein electrophoresis (SDS-PAGE) analysis for Western blot determination of differential markers between EV and solution fractions. After electrophoresis, the protein was transferred onto nitrocellulose membranes by a wet transfer device. The membrane was incubated with nonfat dry milk in Tris buffer (50 mM) to block nonspecific binding, which was followed by incubation for 2 h with primary antibodies directed against CD9, CD63, CD81, or HSP90 (TA500494, ORIGENE Tech.) at 1:2000 dilutions or antibodies directed against human albumin (GTX27793, GeneTex), actinin-4 (GTX101669, GeneTex), and syntenin (ab133267, Abcam) at 1:1000 dilutions. The differential displays of these proteins are used to differentiate between EVs and solutions by Western blot analysis^24,25^. After washing with Tris buffer to remove unbound antibody, a secondary antibody (goat against mouse IgG conjugated with streptavidin-HRP (horseradish peroxidase) at 1:5000 dilutions was incubated for 30 min with the membranes, and then they were subjected to a chemiluminescence reaction. The specific markers on EVs are also verified by flow cytometry, in which we captured the UEVs by anti-CD63-coated beads (63CB-25, Immunostep, Spain), and measured the expression of exosomal surface markers by anti-CD47 antibody labeled with BV421 (BioLegend, CA), and anti-CD63 antibody labeled with AF488 (BioLegend, CA).

### Measurement of the SASP and defense factors

Milliplex antibody-specific beads were used to capture SASP-associated factors and defense factors in urine EVs and drop through fractions. A standard capture sandwich assay was developed with the Luminex Flow Metrix system (Luminex, Austin, TX, USA) to determine the concentrations of SASP-associated factors and immune defense factors. The antibodies directed against specific SASP-associated factors and defense factors were coupled and pooled into a bead-array set (#HBDP-33K, Milliplex MAP, USA) for this assay. Assays were performed using 50 µl of sample, which were analyzed in our immunology laboratory where we routinely measured immune mediators^26^. In brief, urine EV samples were lysed by a lysing buffer at 1:2 volume ratio (Merck cat#43-040) before adding 25 µl of quality controls and standards to the plate in duplicate. This was followed by adding 25 µl of the magnetic beads conjugated with first antibodies. The plate was sealed, wrapped with foil and incubated with agitation on a plate shaker overnight (incubation time of 18 h) at 4 °C. The plate was washed three times and 25 μl of biotin-labeled second antibody was added to each well. After incubating the plate at room temperature (RT) for 60 min, 25 μl streptavidin–phycoerythrin (PE) was added per well. The plate was sealed, covered and incubated for another 30 min at RT. The plate underwent a series of washes before 150 μl of sheath fluid was added for shaking of 5 min. Concentrations of cytokines and defense factors were measured on the BIO-PLEX 200 instrument with Bio-Plex ManagerTM Software 6.1. Quality control values for each marker were consistently within the range indicated by the manufacturer. The accuracy and precision of the Luminex 100 measurement were reported as having a level of misclassification of microspheres < 0.5%.

### Measurement of mRNA in UEV by quantitative RT-PCR

The UEV derived from young (n = 8) and old adults (n = 8) isolated by an Exosome RNA Column Purification Kit (System Biosciences, SBI) were subject to the RNA amplification (SBI) according to the manufacturer’s instruction. RNA concentration was quantified with the NanoDrop 2000 (Thermo Scientific Inc.). Reverse transcription was performed using the Magic RT master Mix assay kit containing both random and oligo-dT primers with 50 ng input RNA, and incubated at 65 °C for 2 min before adding 0.5 μl of MMLV reverse transcriptase for 1 h at 37 °C. Subsequent qPCR was performed using 5 μl of cDNA, the 10 μl 2X SYBR Green Master Mix, 0.8 μl (10 nM) forward and reverse primers, and DEPC water to make a final volume 20 μl. The qPCR primers used are derived from a previous report^27^ and listed as follows: IL-4 Forward GGTCTCAACCCCCAGCTAGT, Reverse GCCGATGATCTCTCTCAAGTGAT; IL-6 Forward ACTCACCTCTTCAGAACGAATTG, Reverse CCATCTTTGGAAGGTTCAGGTTG; IL-8 Forward ACTGAGAGTGATTGAGAGTGGAC, Reverse AACCCTCTGCACCCAGTTTTC; IFNγ, Forward ATGAACGCTACACACTGCATC, Reverse CCATCCTTTTGCCAGTTCCTC; GAPDH Forward GAGTCAACGGATTTGGTCGT, Reverse GACAAGCTTCCCGTTCTCAG. SYBR Green was used as an intercalating DNA dye for measuring fluorescence by using the standard protocol on the Roche LightCycler 96 machine. Thermal cycling for qPCR was performed as follows: 95 °C for 1 min, 40 cycles of 95 °C for 5 s and 37 °C for 30 s. A single peak in the first derivative of the dissociation curve indicated specificity.

### Data normalization and statistics

Biomarker levels in urine could be affected by urine concentration in different physiological conditions (e.g., fasting or postprandial conditions); accordingly, the comparisons of biomarkers in EVs were done after the normalization with each individual number of EVs measured by NTA. Ten pairs of urine samples derived from young adults and elderly individuals were studied based on a study power of 0.8, an effect size of 0.6 (the difference of a SASP cytokine), and an alpha level of 0.05. To compare whether elders with and without Parkinson’s disease had differences in the SASP and in immune defense factors of their EVs, we enrolled another group of 24 PD patients based on an effect size of 0.4 (to determine the difference of a SASP cytokine between elders with and without PD). Data of age, vesicle number and size are presented with mean and standard deviation (SD), and the data of SASP and defense factors were presented with mean and standard error (SE). Normalization for comparisons of SASP protein and mRNA profiles between the UEVs isolated from young and old adults is made by normalization of individual number of EVs measured by NTA. Mann Whitney U test is used to evaluate the significance because the distribution of the data studied did not reach a normal distribution. We have also submitted the EV separation method and quality control measures at EV-TRACK database (http://evtrack.org/review.php), authorized with the access number “EV-TRACK ID (EV210128)”; and the last name “Yang” for the entry.

### Ethics approval and consent to participation

This study is approved by the Institute Review Board of Mackay Memorial Hospital, Taipei, Taiwan. The study was performed after obtaining the informed consent of each participant. All experiments were performed in accordance with relevant guidelines/regulations of the institution review board at Mackay Memorial Hospital.

### Consent to publish

All the authors have reviewed and approved the submission for publication.

## Results

### Isolation and verification of the urine EVs derived from young and old adults

As shown in Fig. 1A, we subjected 40 ml of urine to centrifugation for 30 min to remove cell debris, which was followed by removal of apoptotic bodies through the use of 1.0 µm and 0.22 µm filters. The separation of EVs from drop through fractions was performed by a 0.03 µm filter. The urine EVs were washed and finally concentrated into 0.2 ml (200-fold concentration) aliquots, which were stored in 10 vials at − 80 °C until studies were performed. To confirm that EVs were exclusively isolated, we subjected the same amount of protein (20 µg) from the EVs and solution fractions derived from young adults to Western blot analyses. We found that urine EVs (UEVs) showed prominent CD9 (molecular weight 26 kDa) expression, but lower CD63 (~ 50 kDa) and CD81 (26 kDa) expression; in contrast, plasma EVs (PEVs) expressed higher CD81 and heat shock protein 90 (HSP, 96 kDa), but lower CD9 expression. There were no exosomal markers (CD9, CD63, CD81 or HSP90) detected in the drop through fractions of PEV and UEV. However, the PEV isolated by ExoQuick contained some contamination of albumin, and the UEV isolated from a series of filtration and washes expressed neglectable contamination of albumin (Fig. 1B). Experiments were next performed to compare the EV characters of CD9, CD63, CD81, HSP90, actinin-4 and syntenin expression between the EVs isolated from young and old adults. The results showed similar expression levels of CD9, CD81, HSP90 and syntenin between both groups, but some extent of higher CD63 and actinin-4 expression in the UEVs derived sfrom the elderly (Fig. 1C). We also employed flow cytometric analyses of CD63, CD9 and CD47 expression to verify specific tetraspanin markers of EVs on UEV. As shown in Fig. 1D, we found that CD9 was co-expressed with CD63-enriched UEVs, but not the CD47, which is over-expressed on EVs derived from cancer cells and involved in cancer metastasis^28^. To visualize the UEVs, we used fluorescent analyses of NTA, gating the size between 30 and 300 nm, to demonstrate that UEVs derived from young and old adults are visualized by anti-CD63 antibody labeled with AF488 fluorescent dye (Fig. 1E).

### Number and size of urine EVs derived from young and old adults

**Figure 2:**
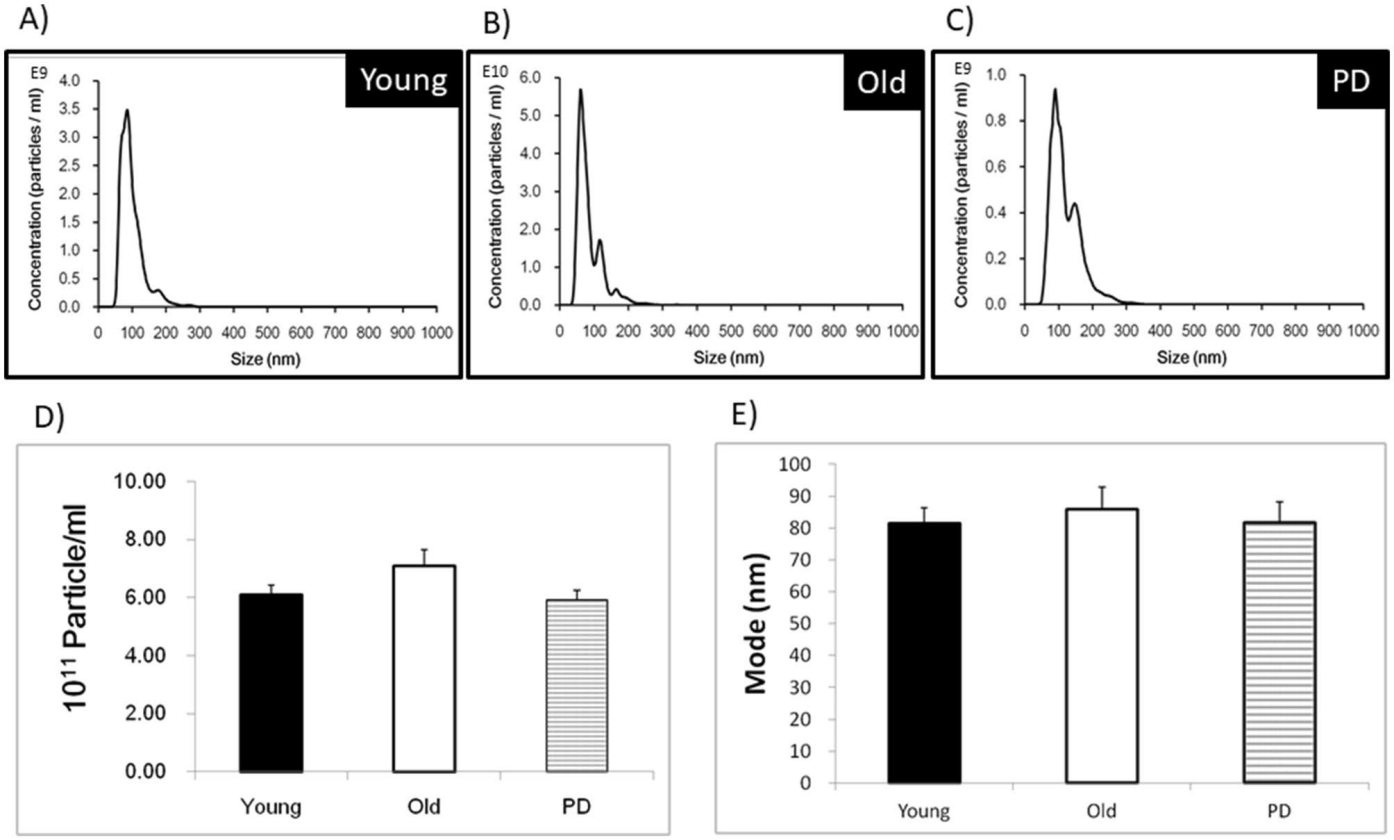
Number and size of urine EVs (UEV) derived from young adults (n = 10) and the elderly without (n = 10) and with PD (n = 24). UEV derived from young adults tend to have a homogeneous profile of EVs (**A**), in comparison to the UEV derived from the elderly without and with PD (**B**,**C**). The vesicle number (mean) at a range between 5.93 and 7.10 × 10^11^ particles/mL after 200-fold concentration of the EVs derived from young and old adults with and without PD was not significantly different among 3 groups although the EVs from PD patients tended to have lower number (**D**). The mean size at a range between 81.6 and 85.9 nm of the EVs derived from young and old adults with and without PD was not significantly different among 3 groups (*p* > 0.05, Mann Whitney U test).

After determining the exosomal markers present in urine EV but not drop through solution, we subjected all the samples of EVs derived from young and old adults with and without PD to measure the EV number and size by NTA. As shown in Fig. 2, we found that UEV derived from young adults tend to have a homogeneous profile of urine EVs (A), in comparison to the UEV derived from old adults without (B) and with PD (C). The vesicle number at a range between 5.93 and 7.10 × 10^11^ particles/ml after 200-fold concentration (backlog, 2.97 and 3.55 × 10^9^ particles/ml, respectively) was not significantly different among 3 groups although the EVs from PD patients tended to have lower number (D). The size at a range between 81.6 and 85.9 nm of the urine EVs derived from young and old adults with and without PD was not significantly different among 3 groups (E) (*p* > 0.05).

### SASP proinflammatory and defense factors in urine EVs and drop-through solutions

Experiments were next performed to differentiate what different contents resided between the EVs and solution fractions. We initially subjected urine EVs (n = 5) and solutions (n = 5) from young adults to measure SASP and defense factors. As shown in Table 2, we found that common SASP mediators such as IP-10, MCP-1, IFNα, IFNγ, IL-4, and IL-10 were mainly present in EVs. In contrast, most of the SASP mediators were undetectable in drop through fraction except small amounts of MCP-1 and IL-10 were detectable in drop through fractions.

**Table 2:** Urine SASP mediators in EVs derived from young adults.

SASP (pg/ml)	EVs (mean ± SE)	Drop through
IL-6	0.1 ± 0.1	ND
IL-8	1.1 ± 0.5	0.1
IP-10	16.9 ± 9.2	ND
MCP-1	86.2 ± 40.6	12.3 ± 5.4
TNFα	ND	ND
IL-12p40	0.1 ± 0.1	ND
IFN-α2	16.9 ± 7.3	ND
IFN-γ	1.3 ± 0.7	ND
IL-10	125 ± 57.8	2.2 ± 0.9
IL-4	2.4 ± 1.7	ND

Data presented are calculated from 10 normal young adults.

*SASP* senescence associated secretory phenotypes, *SE* standard error, *ND* not detectable.

### Different profiles of SASP in urine EVs between young and old subjects

**Figure 3:**
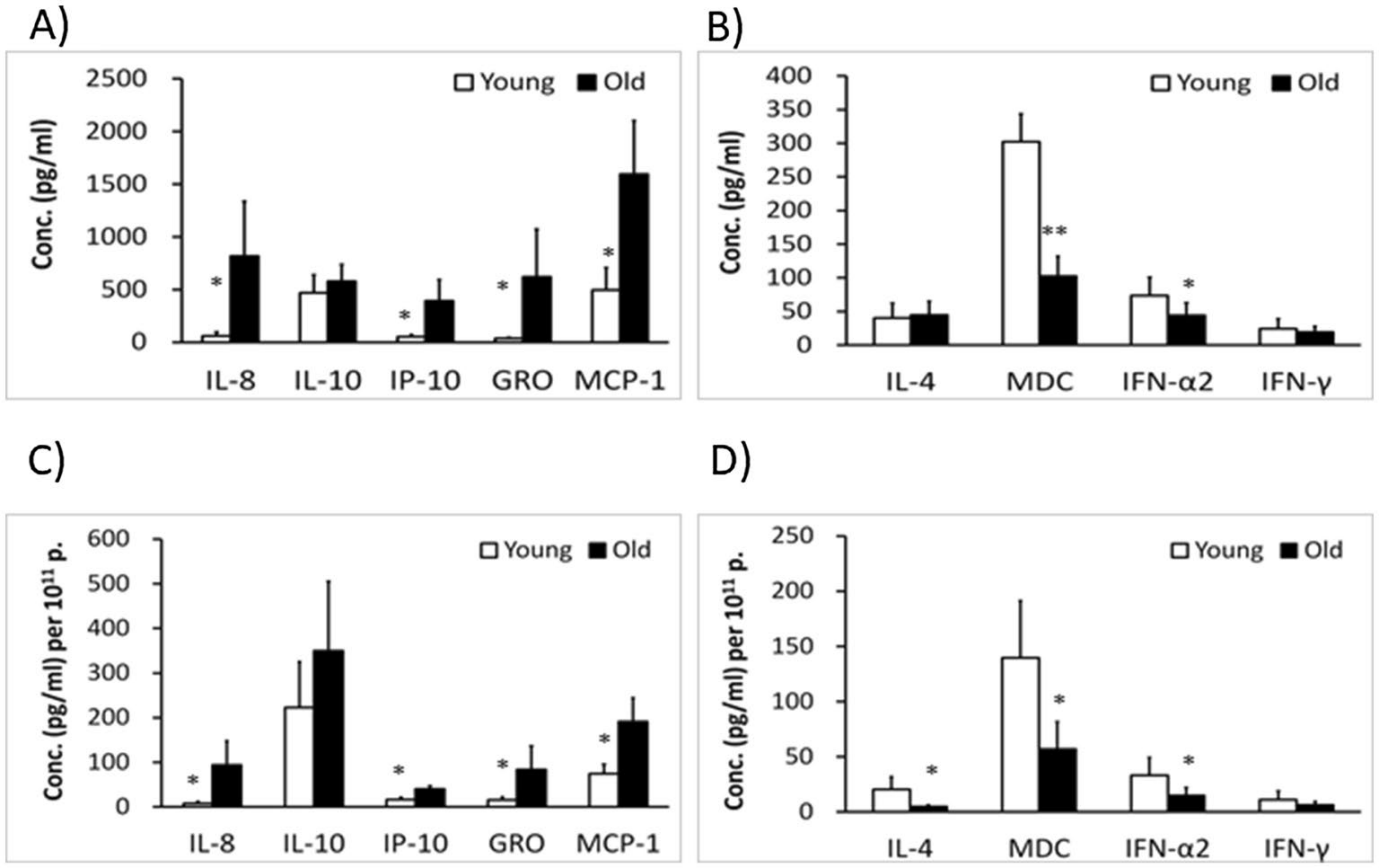
Different SASP and defense factors of urine EVs between young (n = 10) and old (n = 10) adults. UEV derived from the elderly had significantly higher IL-8, IP-10, GRO and MCP-1 levels (**A**) and significantly lower levels of defense factors: IFNα and MDC before normalization (**B**). After the normalization with individual number of EVs, UEV derived from the elderly had significantly higher IL-8, IP-10, GRO and MCP-1 levels (**C**), and significantly lower IL-4, IFNα and MDC levels (**D**). Data are presented with mean ± SE and Mann Whitney U test is used to estimate the significance. *Indicates a significant difference at *p* < 0.05, and **indicates a significant difference at *p* < 0.01.

After identifying SASP mainly present in EVs but not solution, we sought to compare different profiles of SASP between the urine EVs derived from young adults (n = 10) and old adults (n = 10). As shown in Fig. 3A, we found that UEV derived from old adults had significantly (*p* < 0.05) higher IL-8, IP-10, GRO and MCP-1 levels. The higher SASP levels in the elderly remained significantly after normalization of individual number of EVs (Fig. 3C). In contrast, UEV derived from old adults had significantly lower levels of defense mediators IFNα, and MDC before the normalization with individual number of EVs (Fig. 3B). After normalization, older adults had significantly lower IFNα, IL-4 and MDC levels in UEV than young adults (Fig. 3D). Taken together, we showed that the elderly tended to have higher SASP mediators but lower defense mediators in UEV.

**Figure 4:**
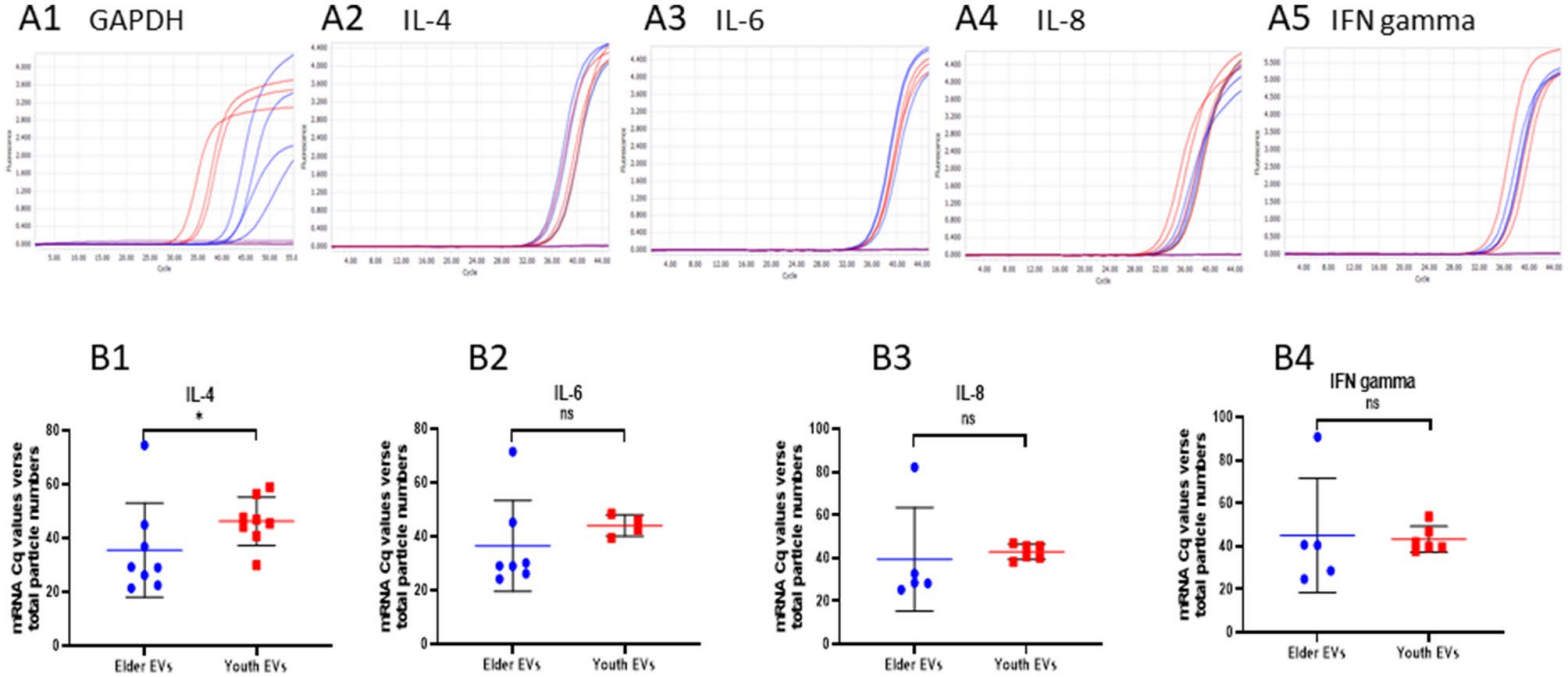
mRNA expression of urine EVs derived from young and old adults. (**A**) qRT-PCR amplification curves of GAPDH and cytokines. The housekeeping gene, GAPDH (**A1**), mRNA expression levels revealed a wide variation among EVs derived from 8 young adults (red lines) and 8 old adults (blue lines) in comparison to those of IL-4 (**A2**), IL-6 (**A3**), IL-8 (**A4**) and IFNγ (**A5**). (**B**) Analyses of cytokines mRNA expression profiles. Results as normalized by individual number of EVs showed a significant lower level of IL-4 (**B1**), but not IL-6 (**B2**), IL-8 (**B3**), or IFN-γ (**B4**), expression in the EVs from the elderly than the EVs from young adults. *Indicates *p* < 0.05 and “ns” indicates no significant difference, analyzed by the Mann–Whitney U test.

Additional studies were performed to investigate whether the higher SASP and lower defense factors of EVs derived from the elderly were compatible with their gene expression (mRNA expression) in EVs. Employing qRT-PCR analyses, we studied the mRNA expression of GAPDH, IL-4, IL-6, IL-8 and IFNγ in the EVs. We found the housekeeping gene, GAPDH, mRNA expression which is usually used as an internal control for its steady state expression, revealed a wide variation among the EVs derived from young and old adults (Fig. 4A1), in contrast to IL-4 (Fig. 4A2), IL-6 (Fig. 4A3), IL-8 (Fig. 4A4) and IFNγ (Fig. 4A5) expression, suggesting the GAPDH mRNA expression in urine EVs is variable depending on age and/or cell origin, and is not a good internal control for comparisons of the cytokine mRNA expression in EVs. We then compared the mRNA expression with the normalization of each individual number of EVs, and found that IL-4 (Fig. 4B1), but not IL-6 (Fig. 4B2), IL-8 (Fig. 4B3) or IFNγ (Fig. 4B4), was significantly lower expression in EVs of the elderly (Fig. 4B1), compatible to the lower IL-4 protein expression (Fig. 3D). Given the facts that EVs are predominantly loaded with rRNA but not mRNA, and contain disproportional proteins in the EVs derived from different cell types^29,30^, it is not unexpected to find the mRNA expressing profiles in urinary EVs were not compatible to the protein expressing profiles at all.

### Different profiles of SASP in urine EVs between elders with and without PD

**Figure 5:**
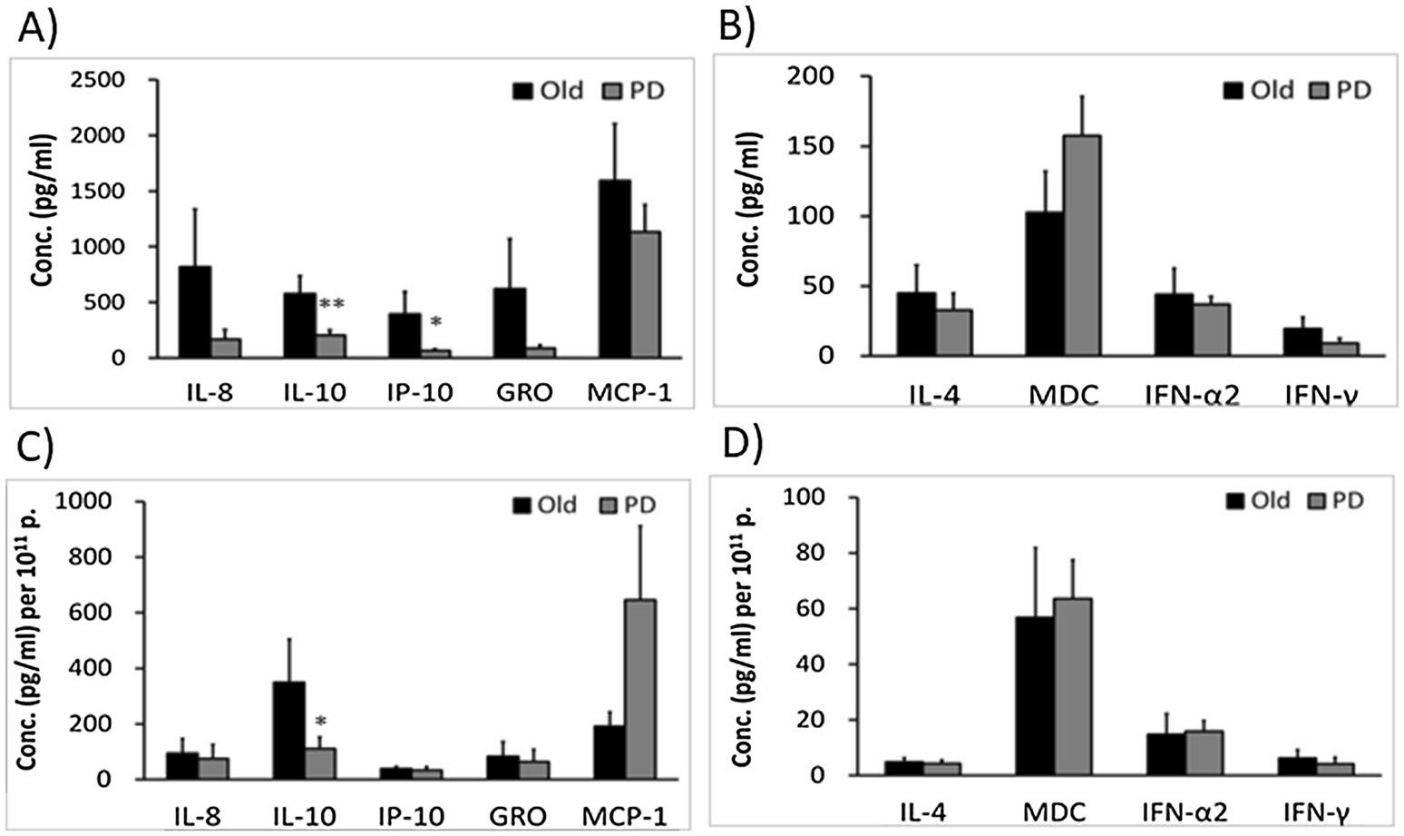
Different SASP and defense factors of urine EVs (UEV) between the elderly with (n = 24) and without (n = 10) PD. UEV derived from the elderly with PD had significantly lower IL-10 and IP-10 levels than the elderly without PD before normalization (**A**). After the normalization with individual number of EVs, the IL-10 levels in EVs from PD patients remained a significantly lower expression (**C**). There were no significant differences on IL-4, MDC, IFNα2 and IFNγ expression between the EVs derived the elderly without and with PD before (**B**) or after (**D**) the normalization with individual number of EVs. Data are presented with mean ± SE and Mann Whitney U test is used to estimate the significance. *Indicates a significant difference at *p* < 0.05, and **indicates a significant difference at *p* < 0.01.

We also sought to differentiate whether elders with and without PD have different profiles of SASP or defense factors. We compared the SASP and defense factors between UEV derived from elders without and with PD. We found that UEV derived from the elderly with PD had significantly lower levels of IL-10 and IP-10 before the normalization (Fig. 5A). After the normalization with individual number of EVs, the IL-10 levels remained significantly lower in the EVs derived from PD patients (Fig. 5C). In contrast, the IL-4, MDC, IFNα2 and IFNγ levels in UEV were not significant differences before and after the normalization (Fig. 5B,D). This suggests that the EVs from the elderly with PD had similar levels of SASP mediators and defense factors except a significantly lower IL-10 level in comparison to those from the elderly without PD.

## Discussion

Most biomarkers of SASP have been studied in blood or tissue cultures^4,5,31,32^. Few studies have tested urine EVs with a size between 50 and 200 nm. We have demonstrated that most of the SASP factors in urine are present in EVs rather than urine solutions. We also found that healthy elderly individuals had significantly higher levels of SASP factors than young adults. More importantly, we found that IFNγ, IL-4 and MDC levels were lower in urine EVs derived from elderly patients than they were from younger patients, and that the lowest levels of IL-10 were found in elderly patients with PD.

Biomarkers of aging in blood, including SASP-associated factors, have recently been studied in proteomic displays of different human cohorts^31–33^. The proteomic signatures of aging have been attributed to the concentrations of certain proteins that increase with chronological age (r = 0.82–0.94), though the signatures vary depending on analyses of different proteins included^33^. However, the protein biomarkers of aging are rarely replicable in different cohort studies or in different experimental aging models^34^. The lack of biomarkers for distinguishing healthy and unhealthy elderly individuals hinder the prediction and prevention of aging. Recent advances in the development of point-of-care testing (POCT) in blood have made biomarkers tested rapidly and useful^35^. However, POCT of biomarkers is mostly performed in blood, serum or plasma, and these samples possess a high complexity due to lipidemia, viscosity and hemolysis, resulting in poor reliability^35^. Moreover, not all biomarkers are in solutions; some are in EVs, making blood POCT difficult. Thus, it would be helpful to be able to identify exosomal biomarkers in the POCT of urine samples, which are less complex, do not require hospital visits to collect and/or do not require painful blood collection procedures. This is particularly beneficial and compassionate in situations of home care or institutional care of elderly individuals.

Some studies have recently identified that cellular senescence in association with secretion of SASP-associated factors plays an important role in aging^31,32^, and transplanted or pre‐existing senescent cells could induce the accumulation of senescent features in surrounding tissues^36^. The mechanisms of biomarkers and molecular pathways involved in aging should be studied, enabling the targeting of a molecule or aging program to eliminate senescence. In addition to blood biomarkers of aging, aging biomarkers in urine have long been studied in cell and soluble macromolecular fractions^7^ but not EVs. Few urine biomarkers based on immunoassays have been approved by the FDA for the prediction of bladder cancers or acute kidney injury^37^. A recent study on biomarkers of renal hypertension identified certain exosomal biomarkers of renovascular hypertension, in which p16 expression of urinary exosomes was elevated in renovascular hypertension patients compared with healthy volunteers, and the levels correlated directly with the renal vein SASP^38^. In an animal study, p16 INK4a expression was found to be correlated with stress-induced premature senescence of renal tubulointerstitial injury^39^. Our study model may pave a novel way to uncover aging biomarkers related to SASP-associated factors and/or immune defense mediators in urinary EVs but not urinary solutions. For instance, a recent clinical trial has demonstrated that senolytic interventions that target senescent cells in humans eliminated senescent cells and decreased levels of SASP-associated factors^40^. We are looking forward to testing whether SASP levels in urine EVs could be used to monitor the effects of synolytic intervention.

In proteomic analyses of elderly individuals with degenerative diseases^41,42^, a number of candidate proteins have been identified as biomarkers of PD in blood and urine, such as calbindin, bone sialoprotein (BSP) and osteomodulin (OMD). Another proteomic marker, leucine-rich repeat kinase 2 (LRRK2), is also suggested to be present in biofluids as a potential biomarker of PD^43^. We analyzed urine exosomal SASP and defense factors in patients with and without PD, and found that PD patients had lower levels of IL-10 in urine EVs. Further studies to validate the urine exosomal biomarkers in elders without and with comorbidities are needed for early prediction and early treatment of degenerative disease.

There are some advantages and limitations of the study on urine exosomal biomarkers of aging. The advantages are that (1) a convenient sampling of urinary EVs can be used to compare the SASP in urine EVs from elderly individuals with and without comorbidities; (2) the data can be quantitatively normalized by individual number of EVs; and (3) a simple device to separate urinary EVs from solutions is useful for identifying aging biomarkers, because biospecimens with less complexity of large volume tissue fluids, such as urine, membrane trapping and/or a series of membrane filtrations could be used to replace combined ultracentrifugation for a rapid diagnosis by point of care device. The limitations are that (1) the urinary EVs are heterogenous and the methods for isolating heterogenous EVs are not standardized yet; (2) the study sample size is relatively small, and (3) the 24 PD patients studied are not homogeneous in the severity or stages. The 24 PD patients recruited from two sick friend associations with definite diagnosis of PD and aged between 60 and 74, with a little higher male to female ratio (13/11). Further studies are needed to investigate whether the aging biomarkers in urine EVs are derived from brain, circulation or regional kidney system, and whether the elderly with and without PD have different urinary exosomal biomarkers beyond SASP-associated factors by another validation with larger sample size and classification of disease severity and stages.

## Conclusions

This study used a simple device to separate urinary EVs from solution for comparisons of SASP and defense mediators between young adults and the elderly with and without PD. Results from this study indicate that aging signature is present in EVs circulating to urine and the signatures include higher inflammatory mediators and lower defense factors. Our study model has paved a novel way to discover aging biomarkers related to SASP mediators and/or immune defense mediators in urinary EVs but not urinary solutions. Further studies to validate the simple method for searching mechanistic aging biomarkers may make prediction of aging and monitoring of anti-senolytic interventions possible.

## Supplementary Information

Supplementary Information.

## Data Availability

The original, uncropped gel images are included in a supplementary file.

## Abbreviations

SASP: Senescence associated secretory phenotypes
PD: Parkinson disease
CSF: Cerebrospinal fluid
PEV: Plasma EVs
UEV: Urinary EVs
Soln: Solution
POCT: Point of care test
