# Supplementary material for: Higher senescence associated secretory phenotype and lower defense mediator in urinary extracellular vesicles of elders with and without Parkinson disease
Source: Sci Rep. 2021 Aug 4;11:15783. doi: 10.1038/s41598-021-95062-y (PMC8339003; doi:10.1038/s41598-021-95062-y)

Supplementary data (original blots) for Figure 1B and 1C

Authors: Shu-hui Yeh, Chia-Hsueh Lin, Yun-Jung Yang, Li-Wei Lin, Chih-Wen Tseng, Kuender D. Yang

Title (Scientific Reports):
Higher senescence associated secretary phenotype and lower defense mediator in urinary extracellular vesicles of elders with and without Parkinson disease

Supplementary Figure 1B. Original blots of CD9, Urine (upper) and plasma (lower)

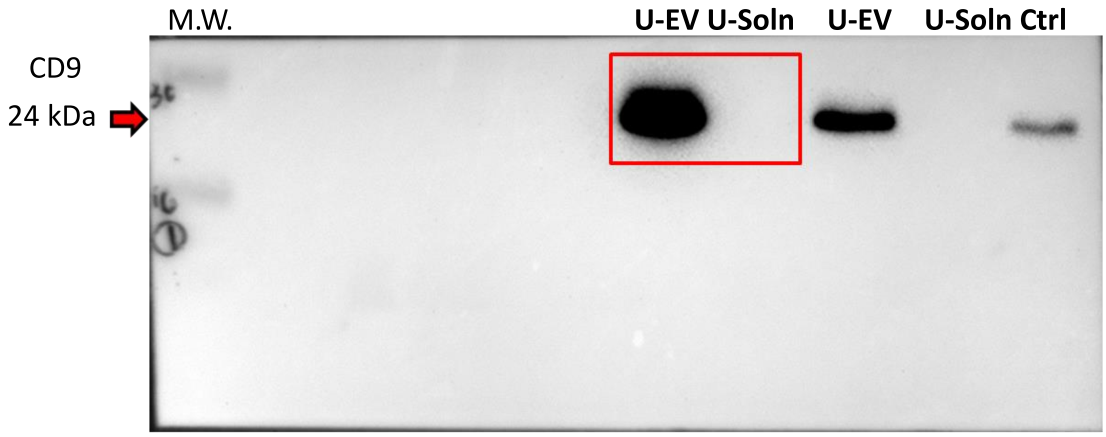

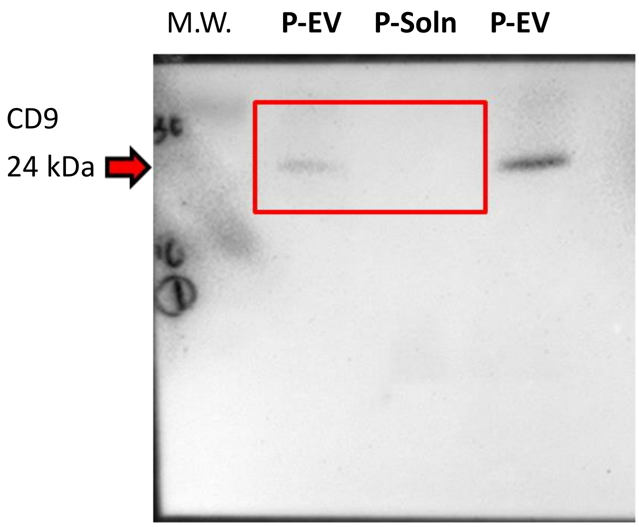

Supplementary Figure 1B. Original blot of CD63, PEV and UEV

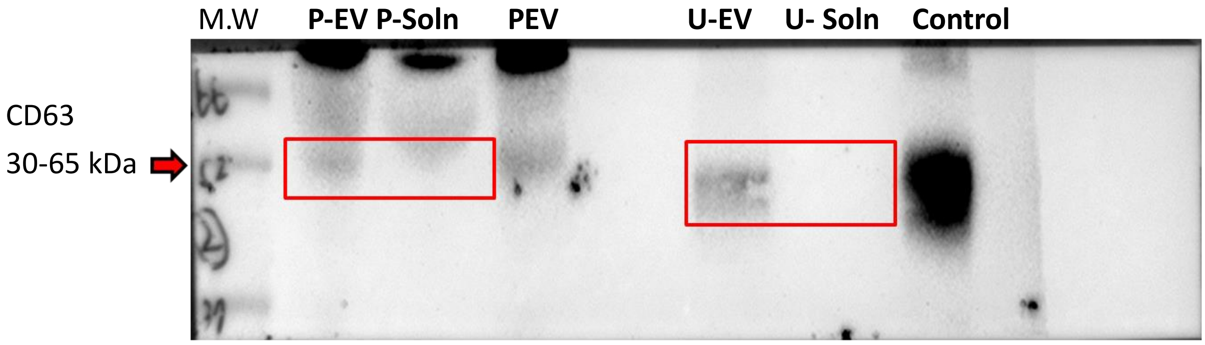

Supplementary Figure 1B. Original blot of CD81 (upper) and HSP90 (lower)

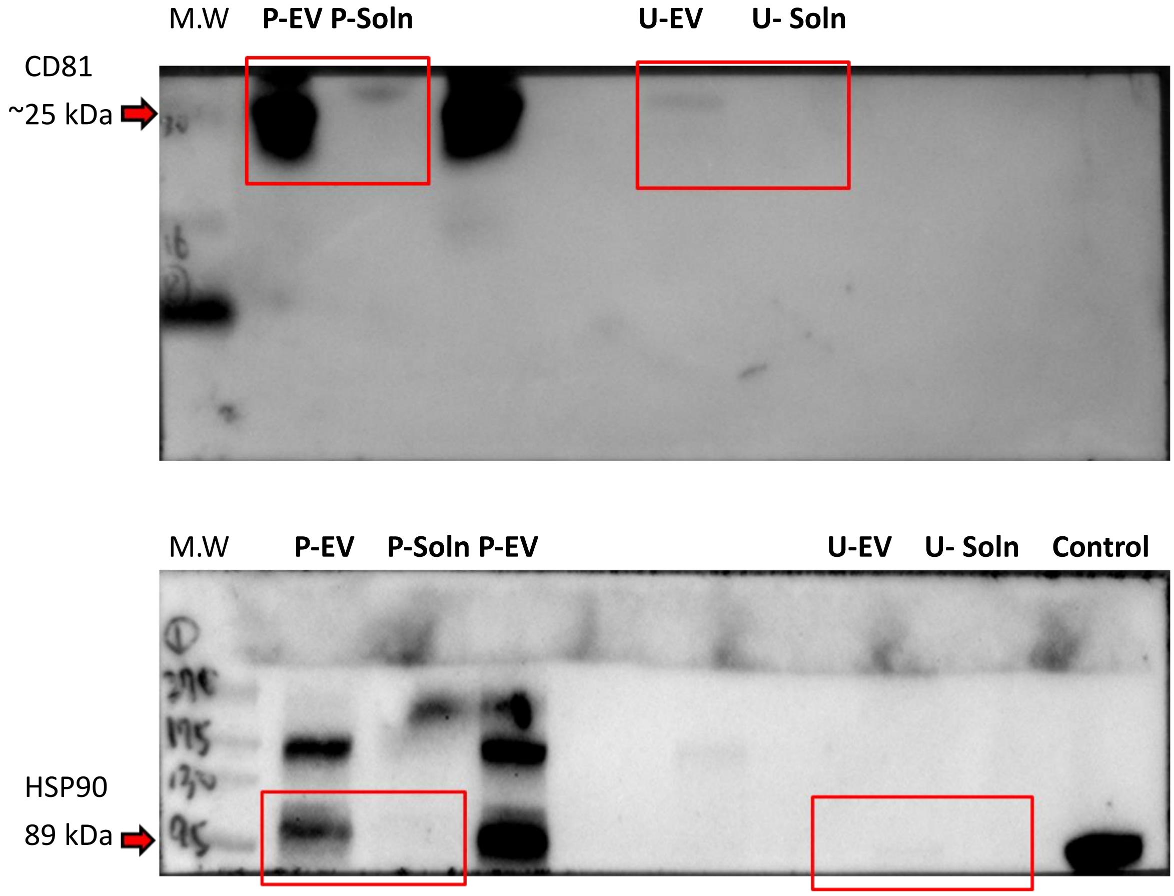

Supplementary Figure 1B. Original blot of human albumin, PEV and UEV

M.W P-EV P-Soln U-EV U- Soln

Albumin
69 kDa

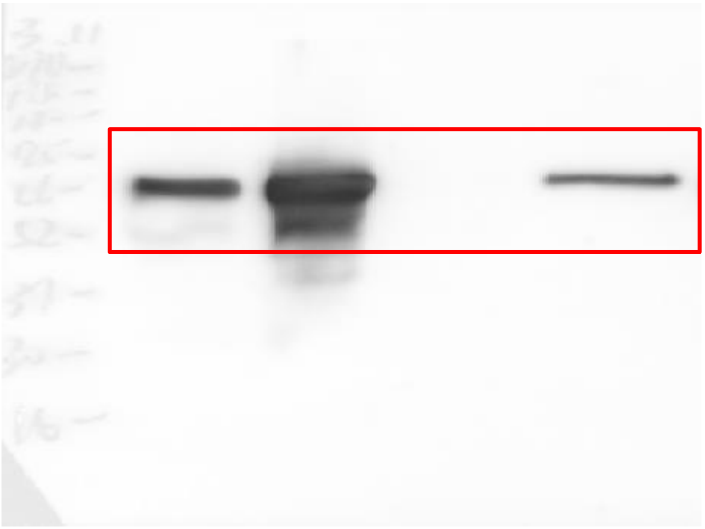

Supplementary Figure 1C. Original blot of CD63, EV (upper) and Soln (lower)

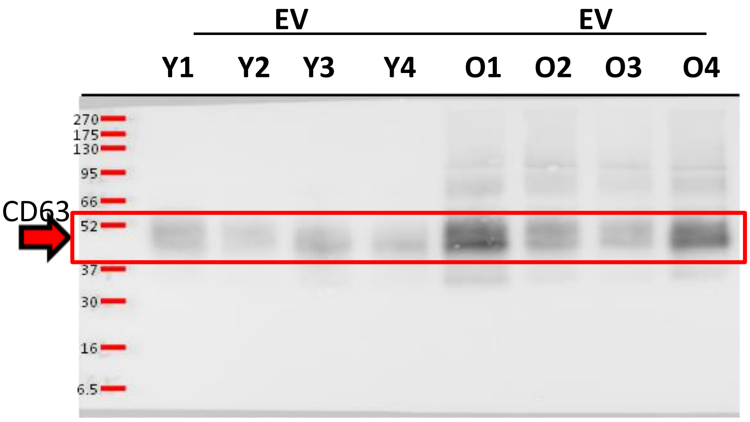

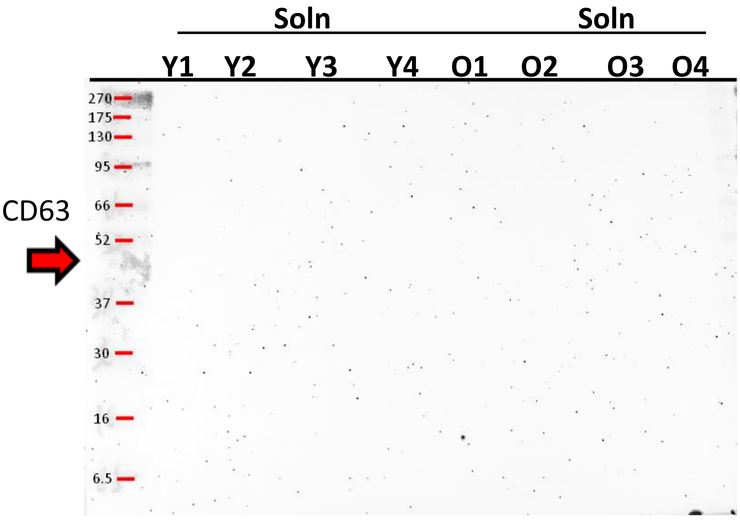

Supplementary Figure 1C. Original blot of CD9, EV (upper) and Soln (lower)

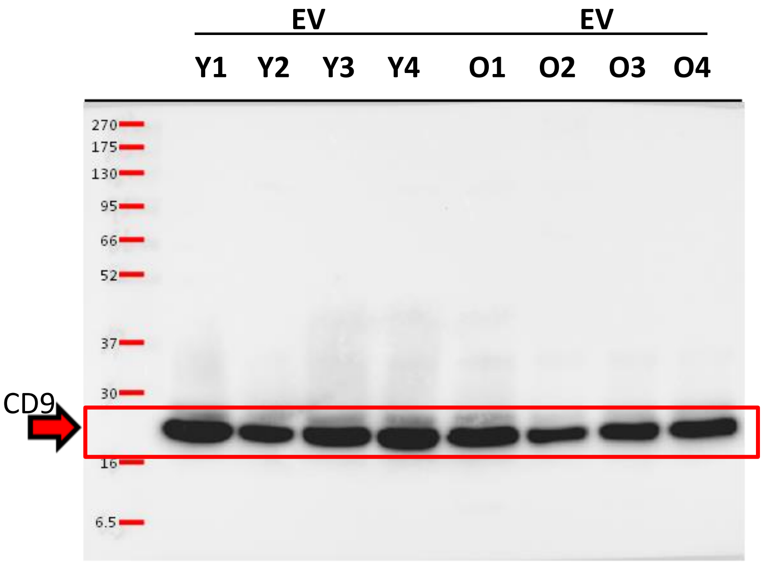

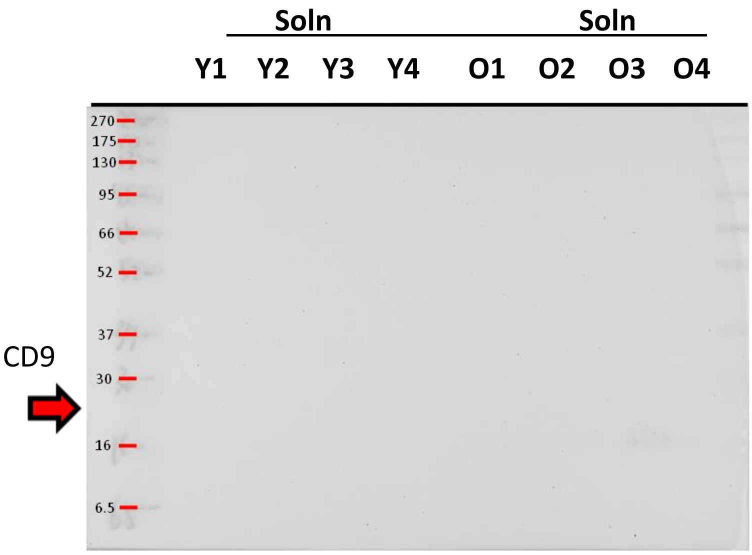

Supplementary Figure 1C. Original blot of CD81, EV (upper) and Soln (lower)

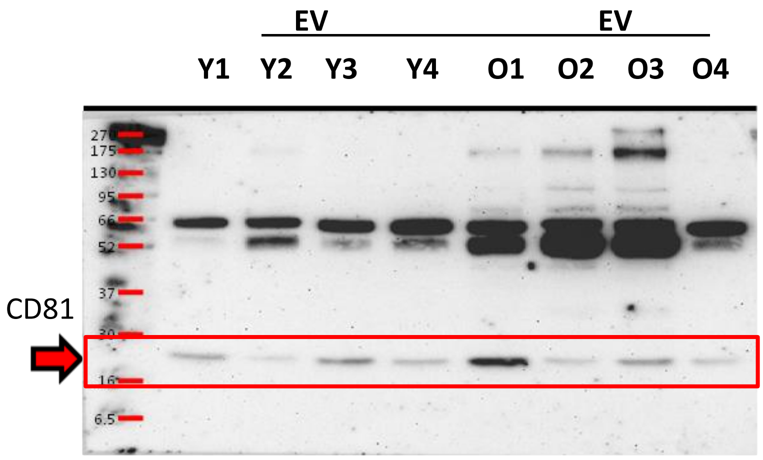

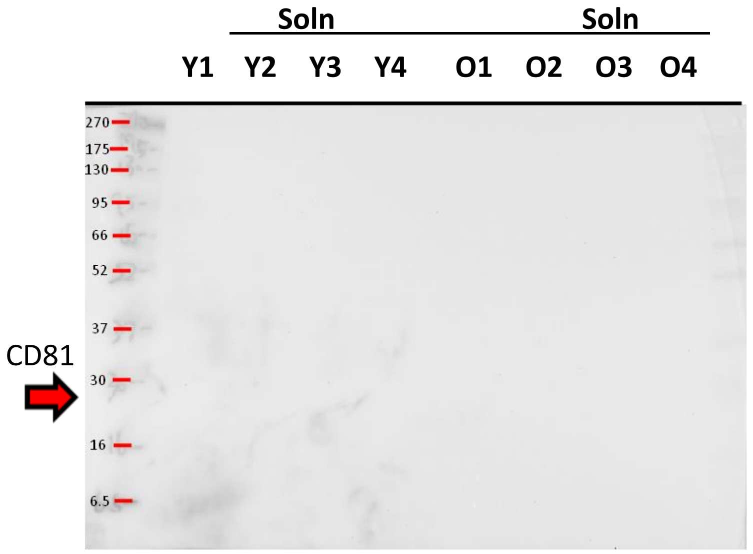

Supplementary Figure 1C. Original blot of HSP90, EV (upper) and Soln (lower)

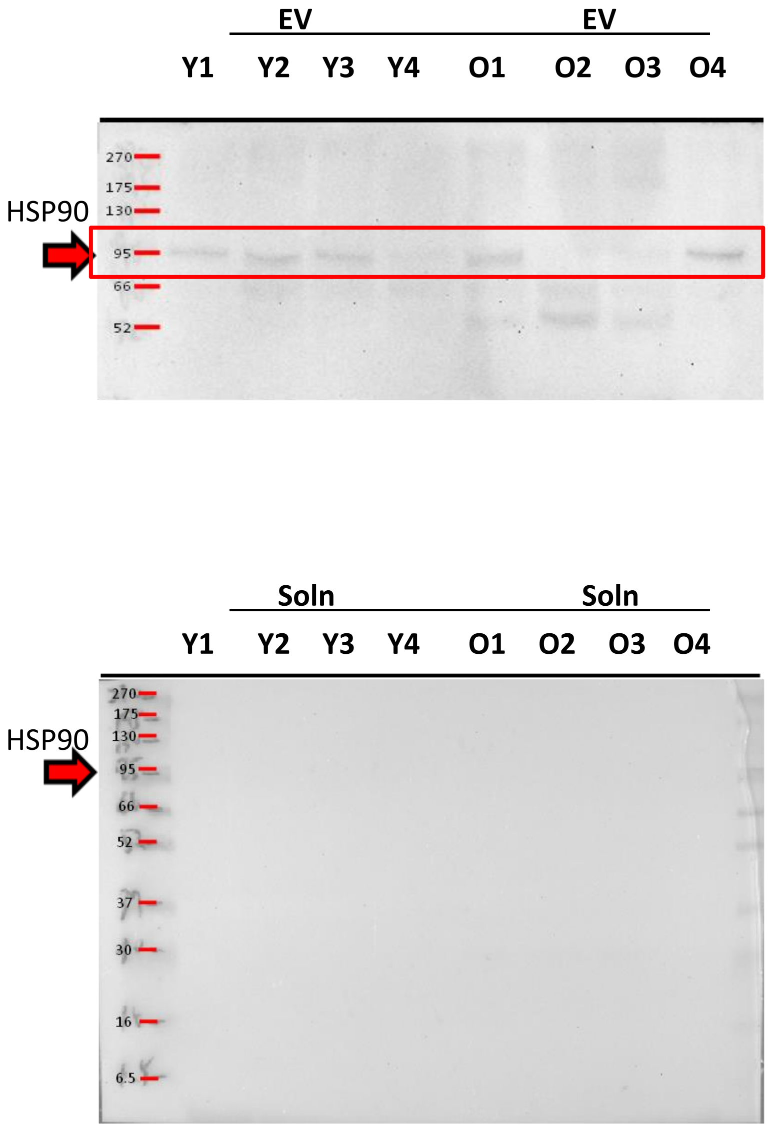

Supplementary Figure 1C. Original blot of Actinin-4, EV (upper) and Soln (lower)

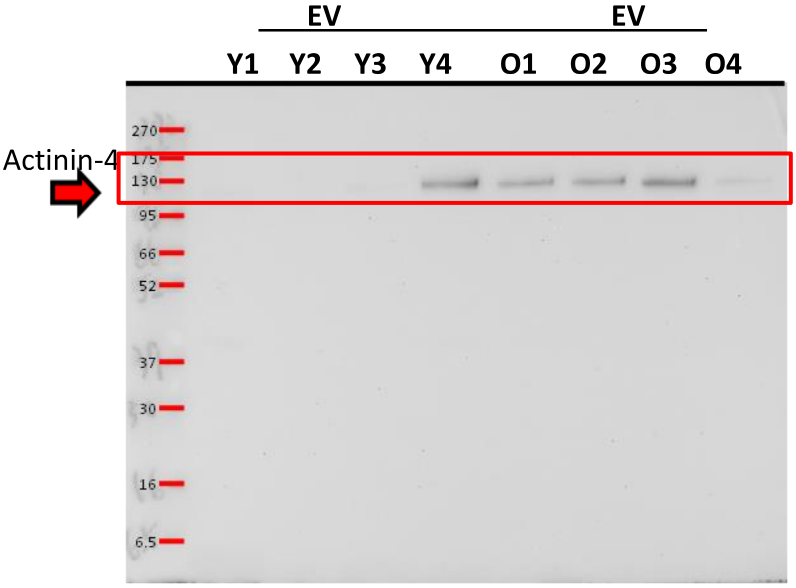

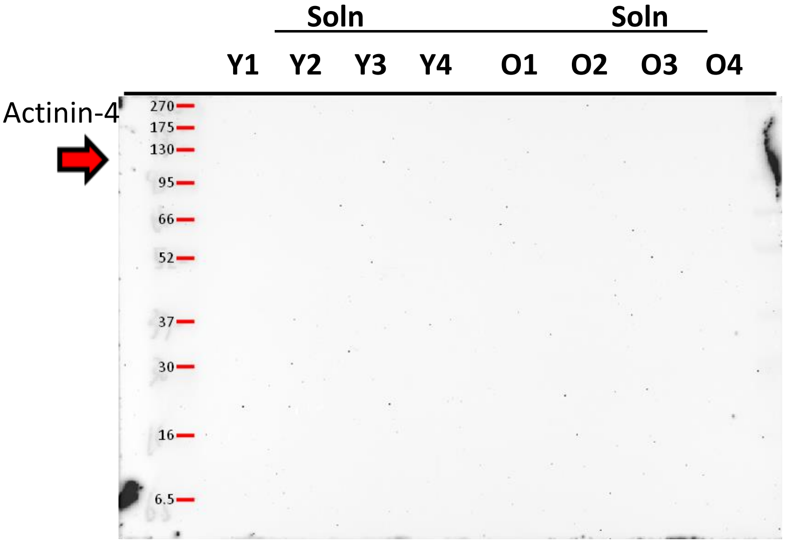

Supplementary Figure 1C. Original blot of Syntenin, EV (upper) and Soln (lower)

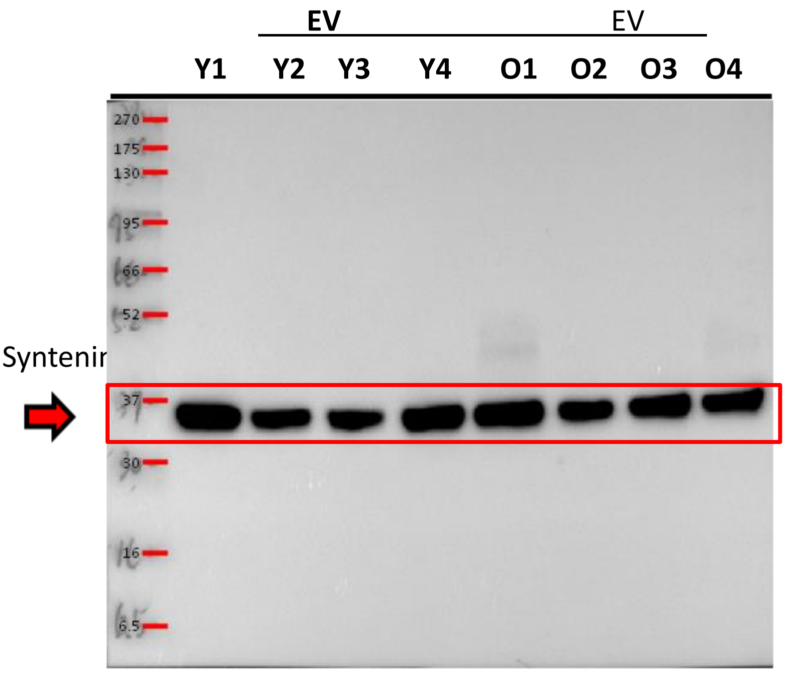

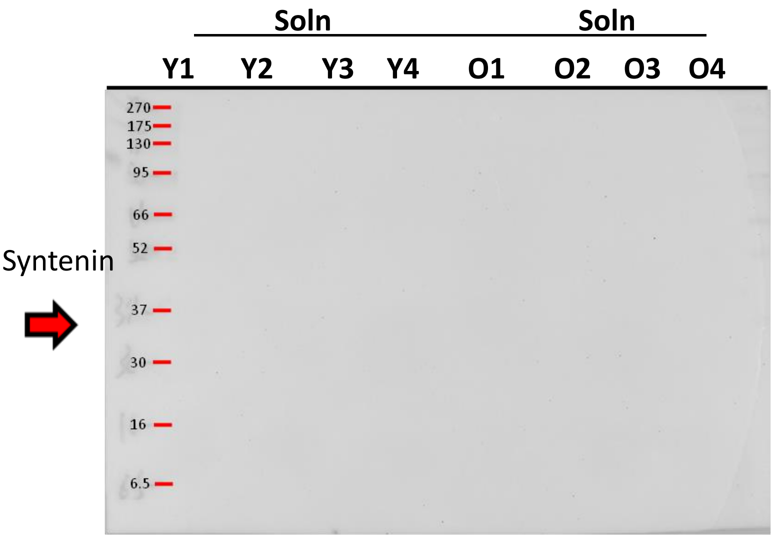

Supplement: Supplementary file 1 — Supplementary Information. [file 41598_2021_95062_MOESM1_ESM.pdf]
